# Switching to an Intravitreal Dexamethasone Implant after Intravitreal Anti-VEGF Therapy for Diabetic Macular Edema: A Review

**DOI:** 10.3390/life14060725

**Published:** 2024-06-03

**Authors:** Livio Vitiello, Giulio Salerno, Alessia Coppola, Ilaria De Pascale, Giulia Abbinante, Vincenzo Gagliardi, Filippo Lixi, Alfonso Pellegrino, Giuseppe Giannaccare

**Affiliations:** 1 Eye Unit, “Luigi Curto” Hospital, Azienda Sanitaria Locale Salerno, 84035 Polla, SA, Italy; giuliosalerno@hotmail.it (G.S.); alessiacoppola330@gmail.com (A.C.); idepascale@outlook.it (I.D.P.); giulia.abbinante@gmail.com (G.A.); v.gagliardi@aslsalerno.it (V.G.); al.pellegrino@aslsalerno.it (A.P.); 2 Eye Clinic, Department of Surgical Sciences, University of Cagliari, 09124 Cagliari, CA, Italy; f.lixi0106@gmail.com (F.L.); giuseppe.giannaccare@gmail.com (G.G.)

**Keywords:** dexamethasone implant, diabetic macular edema, diabetic retinopathy, intravitreal injections

## Abstract

Among working-age people, diabetic retinopathy and diabetic macular edema are currently considered the main causes of blindness. Nowadays, intravitreal injections are widely acknowledged as a significant milestone in ophthalmology, especially for the treatment of several retinal diseases, including diabetic macular edema. In particular, anti-vascular endothelial growth factor (VEGF) agents are typically the first line of treatment; however, monthly injections are required, at least, during the loading dosage. Notably, an intravitreal 0.7 mg dexamethasone (DEX) implant (Ozurdex^®^, AbbVie Inc., North Chicago, IL, USA) is considered a legitimate substitute treatment for diabetic eyes that have not responded to anti-VEGF treatment. In fact, clinical trials and real-life studies have demonstrated the effectiveness and safety of an intravitreal DEX implant in treating such conditions over a period of three to six months. For this reason, wisely selecting diabetic patients might be crucial to decreasing the load of injections in clinics and hospitals. The purpose of this review is to analyze the available scientific literature to highlight the benefits, efficacy, and clinical criteria for choosing whether to switch from intravitreal anti-VEGF therapy to an intravitreal DEX implant in diabetic macular edema.

## 1. Introduction

Nowadays, the most prevalent cause of visual impairment in people of working age is diabetic macular edema (DME) [1]. Type 1 and type 2 diabetes mellitus (DM) is associated with a global prevalence of DME of 45.3% in North America and 11.4% in Europe [2]. While DME can happen at any stage of diabetic retinopathy (DR), it usually presents in cases of mild-to-moderate severity [2], with macular retinal thickening and intra- and subretinal fluid buildup as its characteristic features. In terms of pathophysiology, chronic hyperglycemia increases vascular endothelial factor (VEGF) in both DME and DR, which also increases vascular permeability and angiogenesis [2,3,4,5]. Moreover, vascular permeability and edema are further facilitated by inflammatory mediators, which are crucial to the pathogenesis of DME [6,7,8,9].

Anti-VEGF agents like aflibercept and ranibizumab or the newer brolucizumab and faricimab are the first-line therapy in cases of DME; on the other hand, intravitreal corticosteroids like dexamethasone or fluocinolone acetonide implants are usually used as a second-line strategy. In certain situations, laser treatment and pars plana vitrectomy are further therapeutic possibilities to be considered. Several clinical trials have shown conflicting results on the efficacy of anti-VEGF intravitreal injections in the setting of DME [10,11,12]. It is still unclear why there is such a wide range of pharmacological responses. It appears that hypoxia, inflammation, hyperpermeability, and angiogenesis are the main causes of DME, making it a complex and multifaceted disease; in addition, there may be a buildup of extracellular and intracellular fluids. Nevertheless, not all extracellular fluid is vasogenic; in fact, some fluid is reoriented and accumulates through Müller cells due to inflammation and changes in aquaporin expression.

Several clinical studies have indicated that patients with DME, particularly those with low to normal VEGF levels and elevated levels of inflammatory markers in the blood and anterior chamber, such as ICAM-1, IL-1β, IL-6, and IL-8, do not react appropriately to anti-VEGF therapy [13]. Furthermore, antiangiogenetic drugs generally appear to have a limited effect on long-term and chronic DME [14]. Given its important role, treating inflammation in DME may thus be beneficial for patients who are not responsive to anti-VEGF agents. Considering this aspect, numerous biomarkers have been found to predict therapy response based on the optical coherence tomography (OCT) evaluation [15,16]. For this reason, these biomarkers have the potential to guide an early switch to corticosteroid treatment, as well as individual treatment decisions.

In particular, the sustained-release intravitreal 0.7 mg corticosteroid dexamethasone (DEX) implant (Ozurdex^®^, AbbVie Inc., North Chicago, IL, USA), which contains a biodegradable capsule made of glycolytic and lactic acid polymers, has been demonstrated to be effective in treating DME for three to six months [17,18]. Moreover, it has also been demonstrated that an intravitreal DEX implant is able to suppress inflammation by blocking a number of inflammatory cytokines, which lessens capillary leakage, inflammatory cell migration, edema, and fibrin deposition [19].

The aim of this review is to illustrate the available evidence in the literature about the switch to corticosteroid therapy with an intravitreal DEX implant in patients with DME already treated and not responsive to therapy with intravitreal anti-VEGF agents while also focusing on the clinical and imaging parameters that can guide this therapeutic decision.

## 2. Definition of Persistent and Refractory DME

A patient is categorized as a poor responder and DME is deemed resistant to therapy if the macula is persistently thickened and there is little to no functional change. When categorizing a patient as such, it is important to keep in mind that the precise meaning of limited therapeutic response and poor response is variable. In fact, in post hoc assessments of DRCR.net and VISTA/VIVID data, persistent DME was identified as a central subfield thickness (CST) of more than 250 microns for over six months despite monthly anti-VEGF injections [20]. Conversely, other clinical studies classified DME as refractory when the CST decreased by less than 50 microns or by less than 10% following three monthly anti-VEGF intravitreal injections or when the best-corrected visual acuity (BCVA) worsened [21,22,23]. A panel of experts suggested classifying patients as treatment-resistant to anti-VEGF if, following three consecutive anti-VEGF intravitreal injections, they did not exhibit a reduction of less than 10% CST, a decrease of less than 20% in CRT, or a BCVA increase of more than five Early Treatment Diabetic Retinopathy Study (ETDRS) letters [24]. In light of these disparate definitions, it is crucial to develop a general consensus on the definition of therapy-refractive DME and poor treatment response in order to provide broad recommendations for when and how to change DME treatments. Furthermore, depending on patient characteristics and biomarker profiles, it may be advisable to initiate second-line therapy first in certain circumstances. For this reason, the best initial treatment decision will mostly depend on the use of clinically accessible biomarkers, and algorithms for when to begin therapy and how and when to switch are also required for the best DME management.

## 3. Main Morphological OCT Biomarkers in DME

### 3.1. Intraretinal Cysts

In DME, intraretinal cysts can be considered the OCT hallmark. In particular, cysts indicative of intraretinal fluid buildup can be identified on OCT by their size, reflectance, and placement within the retinal layers, and the underlying etiology of macular edema can be distinguished in part by the cyst’s location [25,26]. In fact, contrary to fluid buildup in the outer nuclear layer (ONL), which is mostly caused by focally leaking microaneurysms, it would appear probable that intraretinal cysts in the inner nuclear layer (INL) caused by widespread artery leakage are more sensitive to anti-VEGF or corticosteroids [27]. Another crucial factor is the size of the cysts [28]; in fact, large cysts are characterized as 250-micron horizontally wide foveal cystoid voids [27] and are associated with increased central subfield thickness, widespread macular edema, macular ischemia, and a higher frequency of outer retinal damage [29]. Consequently, it should come as no surprise that the ONL cyst diameter is a significant predictor of treatment response and functional gains while receiving anti-VEGF agents [29]. Several studies demonstrated that switching to a DEX implant improved BCVA in DME with large parafoveal cysts that were resistant to anti-VEGF therapy [30,31]. Moreover, larger cysts also appear to be linked to chronic DME and a longer course of the disease, and, compared to anti-VEGF treatment, intravitreal corticosteroid therapy is more beneficial for treating chronic DME [32]. In conclusion, chronic DME is linked to large cysts with scarce retinal tissue remaining, and intravitreal corticosteroid therapy may be advantageous for these patients.

### 3.2. Central Subfield Thickness (CST)

The primary criterion for defining and monitoring DME while receiving therapy is still CST. Macular edema is often defined as a CST > 250 microns along with retinal thickness. CST, which is defined as the mean retinal thickness of the center 1 mm on OCT, is used to assess therapy response, disease activity, and progression. However, macular thickness is neither a valid indicator of future functional outcomes nor a good predictor of them. In addition, CST is also not helpful in predicting the morphological response to anti-VEGF vs. intravitreal corticosteroid therapy. On the other hand, treatment response in DME may be predicted by an early decline in CST. While a CST drop of less than 20% is considered a poor response and is linked to limited functional improvement, a decrease of more than 20% in CST is frequently characterized as a response that translates into a large BCVA improvement of more than 10 ETDRS letters [33]. Changes in CST can also be utilized as an indirect indicator of the effectiveness of intravitreal corticosteroids as a therapy, as larger intraretinal layer cysts in DME are often linked to higher CSTs [34]. Furthermore, higher levels of ICAM-1 and sICAM-1, which are responsive to intravitreal corticosteroid therapy but not anti-VEGF treatment, have been associated with a larger macular volume and CST [35]. In conclusion, CST might not be a powerful enough OCT parameter on its own to direct treatment decisions. Intravitreal corticosteroid therapy may typically be seen as advantageous in advanced DME, as a larger CST is linked to large cysts, which are again a sign of chronic DME [15].

### 3.3. Disorganization of the Retinal Inner Layers (DRIL)

In addition to potentially indicating the disruption of pathways that transmit visual information from the photoreceptors to the ganglion cells, DRIL is a prognostic biomarker that depicts the disarray or destruction of cells within the inner retinal layers, including bipolar, amacrine, and horizontal cells [36]. Moreover, DRIL appears to be a predictive biomarker of visual acuity in eyes with baseline center-involved DME that is not reliant on CST. Remarkably, DRIL has also been linked to external retinal damage in the external limiting membrane (ELM) and ellipsoid zone (EZ) [37]. Along with the resolution of DRIL during treatment, functional improvement also occurs. This shows that, as an early indicator of functional results in clinical trials, this OCT biomarker may have predictive relevance. One strong predictor of the long-term recovery of visual acuity is an early three-month improvement in both DRIL and EZ parameters [38]. As a result, a DRIL change could be a useful and accessible noninvasive biomarker of visual acuity for both therapeutic treatment and scientific investigations [15]. If DRIL does not adequately improve, an early switch from anti-VEGF treatment to intravitreal steroids may be explored. Furthermore, the presence and persistence of DRIL during therapy reflect the chronicity of DME. As a result, a number of researchers have suggested that DEX implants may successfully improve DRIL [15,16,39]. In summary, DRIL denotes the death of cells in the inner retinal layer, which may eventually regenerate. A significant predictor of functional vision improvement is the short-term recovery of DRIL under therapy. An early switch to an intravitreal DEX implant should be taken into consideration if DRIL persists while receiving anti-VEGF therapy.

### 3.4. Hyperreflective Foci (HRFs)

HRFs are classified as distinct, well-circumscribed, dot-shaped lesions 20–40 microns in size that have a reflectivity that is at least as high as the retinal pigment epithelium (RPE) band on OCT [40]. They were believed to be lipid-laden macrophages in DME, indicating the presence of inflammatory activity [41]. An alternative interpretation associated HRFs with resident microglial cells that are originally found in proximity to ganglion cells and other inner retinal layers and become activated. As DME and diabetic retinopathy advance, the inflammatory process extends across the retina due to the action of VEGF and other inflammatory mediators. HRFs migrate outward from the inner retina to the outer retinal layers [40,42]. Not only do HRFs enhance CD14 concentrations, but they also raise IL-1β and IL-6 concentrations, highlighting the inflammatory condition in DME [15,16]. As a marker of advanced leakage activity, HRFs are often detected in the choroid as well as at the retinal level, sometimes in close proximity to intraretinal cysts and microaneurysms [40,43,44,45,46]. In DR, the number of HRFs also serves as a biomarker for the severity of the condition [15,16]. The number of HRFs appears to be a significant predictive biomarker for DME and is generally a poor prognostic factor. Numerous investigations have demonstrated that a poor response to anti-VEGF agents is associated with an increased number of HRFs [15,16]. In conclusion, HRFs are often indicative of increased leakage activity in DME and advanced DR. Regardless of the therapeutic approach or medication being used, their existence is associated with inflammation and indicates a greater need for therapy, as well as a shorter duration of benefit. Intravitreal corticosteroid injections appear advantageous in situations of multiple HRFs, namely in the choroid and outer retinal layers; nonetheless, an earlier and more frequent return of DME is to be anticipated. All things considered, HRFs appear to be among the most significant indicators of a greater response to corticosteroids than to anti-VEGF agents.

### 3.5. Subretinal Fluid (SRF)

SRF buildup is believed to indicate either a breakdown of the external retinal blood barrier, which results from damage to the RPE’s tight junctions, or inadequate removal by a malfunctioning RPE pump [47]. Moreover, reduced retinal sensitivity has been linked to the presence of SRF [48]. SRF serves as a biomarker for functional and anatomic treatment response [49]. Before starting therapy, it is present in around 25–30% of DME patients [11]. There are several published studies exploring the effect of SRF on DME, but they are quite contradictory. In fact, according to some clinical studies, there is no relationship between SRF and the degree of DME [50,51]. Others found a correlation between SRF and increased levels of inflammatory cytokines, worse DME, and a more chronic disease condition [22]. In contrast to anti-VEGF therapy, SRF may also indicate a good response to corticosteroids [52,53]. The latter relationship could be explained by increased levels of inflammatory cytokines like IL-6 and IL-8 and by the persistence of high levels of IL-6 in patients who do not respond well to anti-VEGF treatment [54]. Additionally, it is possible that SRF may not have as great of an influence on its own; hence, its existence should be considered in conjunction with other biomarkers. Additionally, it is possible that SRF is associated with distinct stages of the illness in acute and chronic DME. It is rather difficult to determine whether SRF is a prognostic factor. In DME, subfoveal SRF is typically linked to a decline in visual acuity; however, it appears to react to intravitreal injections of corticosteroids or anti-VEGF. However, whether it is a predictor or a prognostic factor is still unclear. In summary, the effects of SRF remain incompletely understood due to the inconsistent results reported in the literature. The few studies that assessed the effectiveness and success of drugs depending on the presence of SRF found that corticosteroids were more useful than anti-VEGF drugs. SRF appears to be a predictor of a positive response to anti-VEGF treatment in cases of acute DME. On the other hand, in chronic DME, the presence of SRF may indicate a poorer response to the same treatment. In addition, the reflectivity of SRF appears to be correlated with VEGF levels. This could indicate that VEGF may play a role in SRF development, while other studies connected the presence of SRF with proinflammatory proteins, including IL-6 and IL-8.

## 4. Switching from Anti-VEGF Therapy to DEX Implant in DME

One of the most difficult challenges in DME management is to provide the best possible therapy to the patient to promote maximum visual recovery as well as anatomical and functional recovery of the retina in the shortest time. Several clinical studies have tried to analyze which drug represents the ideal treatment for individual patients.

Altana et al. [55] enrolled 231 patients with DME (309 eyes) treated with ranibizumab with a loading phase of three injections (one per month) and aflibercept with a loading phase of five injections (one per month). Subsequently, 20 patients with common signs of inflammation on OCT (SRF, HRF, DRIL, etc.) or with a low response to anti-VEGF therapy, determined by poor improvement in visual acuity or a poor reduction in CST, were switched to DEX implants (after the loading phase with anti-VEGF). The switch resulted in significant improvements in CST and BCVA up to the end of the follow-up period (12 months), with no adverse effects detected. 

The efficacy of the DEX implant was also assessed by Totan et al. in 2016 in 30 eyes that had chronic DME and were resistant to at least three bevacizumab injections [56]. At one and three months following the injection, there were significant improvements in both BCVA and CST; however, these benefits did not last over time and tended to decline between the third and sixth months [56]. Similar outcomes were observed in other trials, with around 25% of patients showing improvements in their BCVA six months after the switch [57]. 

These outcomes were also consistent with previous studies by Shah et al. [58], who compared the improvement in visual acuity at 7 months between intravitreal bevacizumab monotherapy and DEX implant monotherapy for chronic DME, with the DEX implant group experiencing fewer injections and lower CST. Remarkably, an evaluation conducted in a real-world scenario on 110 eyes with refractory DME by Busch et al. [59] revealed that eyes that were switched to a DEX implant at 12 months had improved visual and structural results in comparison to those that continued to receive the anti-VEGF drug. 

Recent results from a comprehensive review and meta-analysis [60] showed that, in resistant DME, the DEX implant was linked to a considerably higher improvement in BCVA and a decrease in CST when compared to anti-VEGF therapy. However, the included studies exhibited a high degree of heterogeneity. 

A recent non-randomized interventional study investigated the possible role of a DEX implant in a limited sample of eyes with DME refractory to serial intravitreal injections [61]. Six eyes that had not responded clinically to previous aflibercept injections had DEX implants. The mean BCVA improved and the CST decreased after one year. The authors concluded that the DEX implant showed good anatomic improvement and moderate visual improvement in eyes with DME that were resistant to repeated intravitreal injections [61]. 

Demir et al. [62] analyzed 68 eyes of 68 patients diagnosed with DME who were treated with ranibizumab (3 to 6 injections) but had a poor anatomical response. Of these, 34 patients were switched to a DEX implant after 3 months, while the remaining 34 were switched to a DEX implant after 6 months. Both groups showed improvements in both visual acuity and CST, with comparable results, but the authors suggested switching to a DEX implant as soon as possible if there is a poor response to treatment with ranibizumab. 

Ruiz-Medrano et al. [63] reached the same conclusion, stating that using a DEX implant improved functional results in eyes that did not respond properly to anti-VEGF treatment following three injections. Furthermore, the DEX implant considerably improved anatomical results even in eyes that had undergone more than three anti-VEGF injections [63]. 

In the AUSSIEDEX study [64], early-switch patients had better BCVA (on average) than late-switch patients after 52 weeks, supporting the hypothesis that early treatment with a DEX implant may improve functional outcomes in patients with diabetic macular edema. Furthermore, the results of this study are consistent with data already reported in the literature [65,66,67,68,69], wherein the early switch to a DEX implant in patients who do not respond to anti-VEGF therapy provides better functional results. 

Finally, in their study, Scorcia et al. [70] examined the functional and anatomic results in the eyes of DME patients who received a full anti-VEGF loading dose with aflibercept versus those who were switched to an intravitreal DEX implant following an incomplete anti-VEGF treatment regimen during the 2019 coronavirus disease pandemic. Based on their findings, it appears that patients who were unable to finish their anti-VEGF loading dose during the pandemic period could benefit greatly from DEX implants in terms of both functional and anatomic clinical outcomes. In fact, individuals who were unable to complete the anti-VEGF loading dosage had considerably better functional and anatomic results when using the DEX implant [70].

All of these clinical studies are briefly summarized in Table 1.

## 5. Discussion

**Figure 1:**
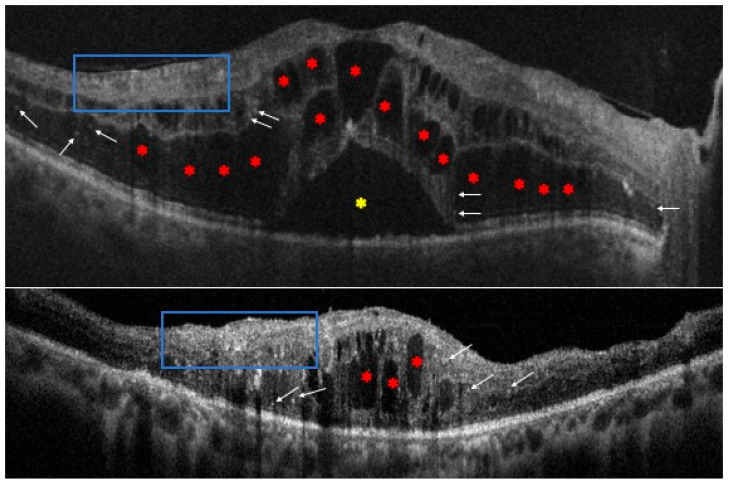
Optical coherence tomography scans of patients with diabetic macular edema. It is possible to observe some of the main morphological biomarkers: intraretinal cysts (red asterisks), subretinal fluids (yellow asterisk), hyperreflective foci (white arrows), and disorganization of the retinal inner layers (blue boxes).

This review aims to analyze the available literature regarding the criteria for switching from intravitreal anti-VEGF therapy to an intravitreal DEX implant in patients suffering from DME while also focusing on OCT parameters that can guide this clinical choice or identify naive patients in whom corticosteroid therapy could represent the first-line therapy (Table 2) (Figure 1). 

Due to the multifactorial nature of DME, some patients may respond better to one treatment approach while others may respond better to the other(s) [71,72]. For this reason, corticosteroids are an essential element of our treatment armamentarium since there is strong evidence that inflammatory proteins are crucial in DME pathogenesis. The anti-inflammatory impact of corticosteroids is really produced through a variety of mechanisms, including a decrease in inflammatory mediator and adhesion protein production and a reduction in VEGF levels, all of which contribute to its multifactorial mechanism of action [73]. In addition, based on their involvement in the development of diabetic retinopathy, several biomarkers for this clinical condition identifiable in local tissues or in the systemic circulation may serve as potential indicators of the underlying pathological processes and as indicators of the response to treatment [74]. They have been measured in the aqueous humor, blood, vitreous, retina, and, more recently, tears [74]. In particular, tumor necrosis factor-alpha (TNFα), IL-1β, IL-6, IL-8, monocyte chemotactic protein-1, and chemokine ligand 5 are the most widely used pathological biomarkers to evaluate the progression and clinical management of diabetic retinopathy, although further clinical studies are needed to better understand their usefulness in clinical practice [74].

Considering the involvement of the above-mentioned molecules in DME pathophysiology, retinal ganglion cells (RGCs) were demonstrated to be impaired in hyperglycemic conditions due to the presence of high levels of cytokines, particularly IL-1β, IL-6, and TNFα. The intravitreal DEX implant has been shown to reduce their concentrations, improving RGC survival in vitro [75]. Moreover, in a rat model of ocular excitotoxicity injury, the intravitreal injection of microparticles containing dexamethasone, vitamin E, and human serum albumin demonstrated the preservation of normal retinal function on electroretinography and the protection of RGCs, confirming the neuroprotective properties of dexamethasone [76].

For all of these reasons, corticosteroid application in DME treatment might be more all-encompassing than anti-VEGF drugs, which only address a portion of the angiogenetic cascade [77]. The Euretina recommendations state that corticosteroids are mostly second-line treatments in DME and should only be given to patients who do not adequately respond to anti-VEGF injections (after three to six injections, depending on each patient’s unique response). Nonetheless, patients with a history of significant cardiovascular events and those who are unable to attend monthly or more frequent appointments are seen as the most suitable candidates for first-line treatment [78]. In particular, patients with high-risk cardiovascular disease, poor compliance, severe macular edema (more than 500 microns), a history of cataract surgery or vitrectomy, or scheduled cataract surgery are advised to use the DEX implant as first-line treatment [79,80]. Other alternative treatments for refractory DME are represented by the use of different intravitreal steroids or subthreshold micropulse laser (SML) therapy. A fluocinolone acetonide (FAc) intravitreal implant (Iluvien, Alimera Sciences Limited, Aldershot, UK) containing 190 μg of FAc, which is continuously released into the vitreous body for up to 36 months, is considered a cost-effective and time-saving approach to managing DME. Its use is approved by the FDA for DME patients who have previously received a DEX implant without experiencing a significant rise in IOP. However, it should be noted that a high percentage of patients can present increased IOP after Iluvien, necessitating medical therapy or requiring glaucoma surgery [81]. Moreover, triamcinolone acetonide (TA) injections are another valid treatment option for DME refractory to anti-VEGF drugs. Despite the higher risk of complications such as IOP elevation, it has been shown to improve BCVA and to reduce the interval of recurrences of macular edema [82]. Finally, the SML, using various laser wavelengths, aims to stimulate a biological response without causing retinal damage. Specifically, the use of a yellow subthreshold micropulse laser (YSML) has been studied for the management of DME, thus offering a complementary therapy, reducing the economic burden of multiple anti-VEGF injections, and preserving or improving morpho-functional outcomes [83].

Nevertheless, the DEX implant is preferred in cost–benefit analyses [79,84,85,86]. According to international guidelines, the best DME treatment for a diabetic patient will therefore depend on numerous factors, including the patient’s baseline status, the identification of predictive biomarkers, and the patient’s responsiveness to individual treatment [78]. In DME, biomarkers have been utilized to predict possible responses to various therapies as well as to distinguish between generally poor and good responders. Large intraretinal cystoid spaces, higher baseline CST, and more choroidal HRFs are all prognostic of a good treatment response to corticosteroids but are often linked to a limited treatment response [39]. Conversely, the response of other DME parameters, including progressive ischemic maculopathy, appears to be almost the same for both DEX and anti-VEGF [15,16]. In patients who have not received therapy yet, corticosteroid implants have shown acceptable clinical results and a positive safety profile [79]. Following a switch to corticosteroids after anti-VEGF therapy, there have been observed modifications in particular inflammatory parameters in the inner retina, such as decreases in HRFs, DRIL extension, and CST [15]. Concerning the time of switching, a general consensus is still lacking. However, most clinical studies recommend waiting at least until the end of the three anti-VEGF loading doses before making the switch or carefully evaluating the OCT parameters and biomarkers to identify inflammatory components that could favor better clinical results with a DEX implant [87,88]. Regarding the safety profile, the systemic and local adverse effects of intravitreal DEX are somewhat reduced compared to those of other steroids (triamcinolone, fluocinolone). Indeed, its intravitreal route of delivery and its minimum systemic absorption reduce the rate of systemic adverse events. Furthermore, DEX is highly soluble in water, exhibiting faster clearance from the eye compared to other steroids, which can impact its binding properties to intraocular tissues and potentially reduce the incidence of local events. Nonetheless, the affinity of intravitreal steroids for lens and trabecular meshwork cells can lead to complications such as cataract progression and an IOP increase [79]. The latter is usually manageable with topical IOP-lowering medications, with few cases requiring filtration surgery [89]. Additionally, although the higher incidence of cataract development in patients receiving DEX implants compared with anti-VEGF therapy is still discussed, this complication is easily manageable with cataract surgery, with a preference to avoid or postpone the DEX implant in phakic patients [79,90,91].

## 6. Conclusions

The evaluation of morphological biomarkers at the beginning of and during therapy can guide the treatment plan and perhaps influence the decision to switch to corticosteroid treatment early. Biomarkers from retinal imaging might provide more individualized care with improved visual results [92]. Machine learning techniques will soon enable more tailored therapeutic strategies, with the optimal course of action determined by analyzing the significance and interactions of individual features, as well as a vast array of biomarkers. Patients with DME who have biomarkers that indicate a generally negative response to existing treatment choices may benefit from upcoming next-generation medications that inhibit tyrosine kinase or disrupt integrin pathways, for example. Appropriate therapy and the prevention of vision loss in patients with resistant and long-term DME require an early switch to a DEX implant to prevent the permanent loss of retinal cells owing to chronic edema. However, further studies are needed to better understand and standardize the exact time of switching from anti-VEGF treatment to a corticosteroid implant. 

## Figures and Tables

**Table 1:** A summary of the clinical studies analyzing the switch from intravitreal anti-VEGF therapy to an intravitreal DEX implant in DME.

Author (Year)	Reference	Type of Study	Population	Outcomes
Altana et al. (2021)	[55]	Retrospective study	Twenty patients with common signs of inflammation on OCT or with a low response to previous anti-VEGF therapy were switched to an intravitreal DEX implant.	The switch in non-responders resulted in significant improvements in CST and BCVA up to the end of the follow-up period (12 months), with no side effects.
Totan et al. (2016)	[56]	Prospective study	Thirty patients who had chronic DME and were resistant to at least three intravitreal bevacizumab injections were examined at baseline and 1, 3, and 6 months after the switch to an intravitreal DEX implant.	Significant improvements in both BCVA and CST were recorded up to the third month. Macular edema recurrence occurred in 25 eyes at 6 months. IOP increased significantly at 1 week, 1 month, and 3 months but was controlled with topical anti-glaucoma monotherapy.
Kim et al. (2016)	[57]	Retrospective interventional case series	Thirty-five patients were treated with a single intravitreal injection of DEX for refractory DME despite multiple intravitreal bevacizumab injections.	DEX significantly reduced central foveal thickness and choroidal thickness, with BCVA improvement in 26% of eyes at 6 months.
Shah et al. (2016)	[58]	Prospective randomized subject-masked study	Twenty-seven eyes with persistent DME despite more than 3 anti-vascular endothelial growth factor injections within 5 months were switched to an intravitreal DEX implant.	The DEX group achieved a significantly greater reduction in CST compared with the intravitreal bevacizumab monotherapy group, with no recurrent edema at any visit.
Busch et al. (2018)	[59]	Retrospective multicenter, case–control study	Thirty-eight eyes were switched to intravitreal DEX implants after receiving anti-VEGF therapy.	Eyes switched to a DEX implant at 12 months had improved visual and structural results in comparison to those that continued to receive the anti-VEGF drug.
Wilkins et al. (2021)	[61]	Non-randomized interventional study	Six eyes with DME refractory to serial intravitreal injections underwent treatment with intravitreal DEX implants.	The DEX implant appeared effective in eyes with super-refractory DME, resulting in excellent anatomic improvement on OCT, as well as modest visual improvement.
Demir et al. (2020)	[62]	Retrospective study	Thirty-four patients were switched to intravitreal DEX implants after 3 months (early switch), while another thirty-four patients were switched to DEX implants after 6 months (late switch).	Both groups showed improvements in both BCVA and CST, with comparable results, but an early switch is recommendable in cases where there is a poor response to treatment with anti-VEGF.
Ruiz-Medrano et al. (2021)	[63]	Multicenter, retrospective, and real-life case series study	One hundred twenty-nine eyes undergoing treatment with DEX were divided into three groups: I—naïve patients; II—previously treated eyes that received 3 intravitreal anti-VEGF injections before the study (early switch); and III—previously treated eyes that received more than 3 intravitreal anti-VEGF injections (late switch).	By month 12, BCVA and CST had statistically improved in all groups. The improvements were significantly greater in the early-switch group than in late-switch patients.
Mitchell et al. (2023)	[64]	Prospective, open-label, observational, real-world study	Of 143 eyes, 53 (37.1%) and 89 (62.2%) were switched to DEX after 3–6 months (early) and >6 months (late) after anti-VEGF injections, respectively.	The change in mean BCVA from baseline was not significant at week 52. However, the early–late-switch difference in BCVA was statistically significant at week 52, suggesting that early-switch patients had a greater BCVA improvement at week 52 than late-switch patients. The mean CST improved significantly from baseline. No unexpected adverse events were reported.
Lee et al. (2023)	[65]	Retrospective study	Thirty-one patients were included in the DEX switching group after bevacizumab treatment.	DME with large serous retinal detachment and retinal edema may be more effectively treated with the DEX implant than bevacizumab. Accordingly, in the switching group, central macular thickness, inner cystoid macular edema, and serous retinal detachment volume all showed significant reductions after switching to the DEX implant.
Cicinelli et al. (2017)	[66]	Retrospective study	Forty-five patients with DME were switched to an intravitreal DEX implant after three injections of ranibizumab and followed up for 12 months.	After 3 injections of Ranibizumab, 30 eyes (66.7%) had a poor visual response, while 15 eyes had good visual outcomes. Patients with a poor visual response were associated with limited morphological improvement. One month after receiving the DEX implant, only poor responders showed a relevant increase in BCVA and a reduction in central macular thickness in comparison to good-visual-response patients.
Maggio et al. (2018)	[67]	Retrospective interventional case series	One hundred twenty-nine patients were included in the study. After 3 monthly intravitreal anti-VEGF injections, a subgroup of eyes that were unresponsive to the treatment received alternative therapeutic options, including switching to another anti-VEGF drug, an intravitreal injection of DEX, and vitrectomy.	In eyes with a suboptimal response, no significant visual improvement was found when switching to another anti-VEGF treatment. Twenty-four eyes treated with a DEX implant and fourteen with vitrectomy exhibited a significant reduction in central macula thickness with variable functional responses. In these eyes, a better BCVA gain was found in cases with an early change of the treatment strategy.
Hernández Martínez et al. (2020)	[68]	Retrospective study	Of 69 DME eyes, 31 eyes were included in the early-switch group and 38 were included in the late-switch group after intravitreal anti-VEGF treatment.	In the early-switch group, BCVA had significantly increased by month 24, whereas, in the late-switch group, BCVA did not increase. Morphological parameters were improved in both groups, with the proportion of eyes obtaining a CST ≥ 10% being significantly greater in the early-switch group than in the late-switch group.
Busch et al. (2019)	[69]	Multicenter, retrospective study	One hundred ten eyes with treatment-naïve DME and a suboptimal response to a loading phase of anti-VEGF therapy (3 monthly injections) were then treated with further anti-VEGF (n = 72) or initially switched to DEX implant (n = 38).	The beneficial effect of an early switch to the DEX implant in DME non-responders seen at month 12 was maintained in the second year. A later switch from anti-VEGF to steroids still provided significant improvement.
Scorcia et al. (2021)	[70]	Retrospective, comparative study	Of 43 eyes, 23 eyes underwent a complete VEGF loading dose with aflibercept, and 20 eyes were switched to a DEX implant after incomplete anti-VEGF treatment during the coronavirus disease 2019 pandemic.	The mean BCVA and CST had significantly improved by month 4 in both groups. Therefore, the DEX implant could significantly improve both functional and anatomic clinical outcomes in patients who were unable to complete the anti-VEGF loading dose.

OCT: optical coherence tomography; DEX: dexamethasone; BCVA: best-corrected visual acuity; CST: central subfield thickness; IOP: intraocular pressure; VEGF: vascular endothelial growth factor.

**Table 2:** A summary of the main optical coherence tomography morphological biomarkers that could guide the therapeutic choice in cases of diabetic macular edema.

Biomarker	Good Response to Anti-VEGF	Good Response to Steroids
Large intraretinal cysts (more than 250 microns)	NO	YES
Central subfield thickness	NO	NO
Disorganization of retinal inner layers	NO	YES
Hyperreflective foci	NO	YES
Subretinal fluid (acute DME)	YES	NO
Subretinal fluid (chronic DME)	NO	YES

## Data Availability

Not applicable.
